# Effects of Bariatric Surgery on Adipokine-Induced Inflammation and Insulin Resistance

**DOI:** 10.3389/fendo.2013.00069

**Published:** 2013-06-10

**Authors:** Zeynep Goktas, Naima Moustaid-Moussa, Chwan-Li Shen, Mallory Boylan, Huanbiao Mo, Shu Wang

**Affiliations:** ^1^Nutritional Sciences Program, College of Human Science, Texas Tech University, Lubbock, TX, USA; ^2^Department of Pathology, Texas Tech University Health Sciences Center, Lubbock, TX, USA; ^3^Department of Nutrition and Food Sciences, Texas Woman’s University, Denton, TX, USA

**Keywords:** bariatric surgery, inflammation, obesity, insulin resistance, adipokines

## Abstract

Over a third of the US population is obese and at high risk for developing type 2 diabetes, insulin resistance, and other metabolic disorders. Obesity is considered a chronic low-grade inflammatory condition that is primarily attributed to expansion and inflammation of adipose tissues. Indeed, adipocytes produce and secrete numerous proinflammatory and anti-inflammatory cytokines known as adipokines. When the balance of these adipokines is shifted toward higher production of proinflammatory factors, local inflammation within adipose tissues and subsequently systemic inflammation occur. These adipokines including leptin, visfatin, resistin, apelin, vaspin, and retinol binding protein-4 can regulate inflammatory responses and contribute to the pathogenesis of diabetes. These effects are mediated by key inflammatory signaling molecules including activated serine kinases such as c-Jun N-terminal kinase and serine kinases inhibitor κB kinase and insulin signaling molecules including insulin receptor substrates, protein kinase B (PKB, also known as Akt), and nuclear factor kappa B. Bariatric surgery can decrease body weight and improve insulin resistance in morbidly obese subjects. However, despite reports suggesting reduced inflammation and weight-independent effects of bariatric surgery on glucose metabolism, mechanisms behind such improvements are not yet well understood. This review article focuses on some of these novel adipokines and discusses their changes after bariatric surgery and their relationship to insulin resistance, fat mass, inflammation, and glucose homeostasis.

## Introduction

Obesity refers to excess fat mass or adiposity, and is typically defined as a body mass index (BMI) over 30 kg/m^2^ or a waist circumference greater than 94 cm for men and 80 cm for women (Nash et al., 2008). It is a complex multifactorial disease that is positively associated with increased risk and onset of numerous chronic diseases including cardiovascular disease and Type 2 diabetes (Sjostrom et al., 2004). According to the World Health Organization report, one billion of the world’s adult population is overweight and 300 million of them are obese (Mushtaq et al., 2011). One of three adults is obese in the United States (Bhattacharya and Sood, 2011).

The first recommended treatment option for obesity is a low-calorie diet and regular physical activity. However, these lifestyle interventions have low compliance in general and limited effectiveness in severely obese people (Hell and Miller, 2002; Yermilov et al., 2009). Bariatric surgery has emerged as the approach of choice for weight loss among morbidly obese adults with a BMI over 40 kg/m^2^ or those with a BMI over 35 kg/m^2^ and existing metabolic risk factors such as hypertension, diabetes, or hypercholesterolemia (Sjostrom et al., 2004; Kulick et al., 2010).

Recent research has established that obesity is a chronic low-grade inflammatory condition (Herder et al., 2007; Liu et al., 2007; Amati et al., 2010; Balistreri et al., 2010; Park et al., 2010; Thompson et al., 2011). Following bariatric surgery, patients usually experience approximately 30% weight loss as well as decreased overall inflammatory responses (Compher and Badellino, 2008). Concomitantly, there are beneficial changes such as improved insulin resistance, reduced cardiovascular risk, and decreased oxidative stress that are achieved through multiple pathways related to systemic and adipocyte inflammation and adipocyte-derived cytokines (Cancello et al., 2005; Holdstock et al., 2005; Mattar et al., 2005; Vazquez et al., 2005; Poitou et al., 2006; Iannelli et al., 2009, 2010; Boesing et al., 2010; Butner et al., 2010; Hofso et al., 2010; Joao Cabrera et al., 2010; Murri et al., 2010; Kalupahana et al., 2011, 2012).

## Bariatric Surgery

**Figure 1:**
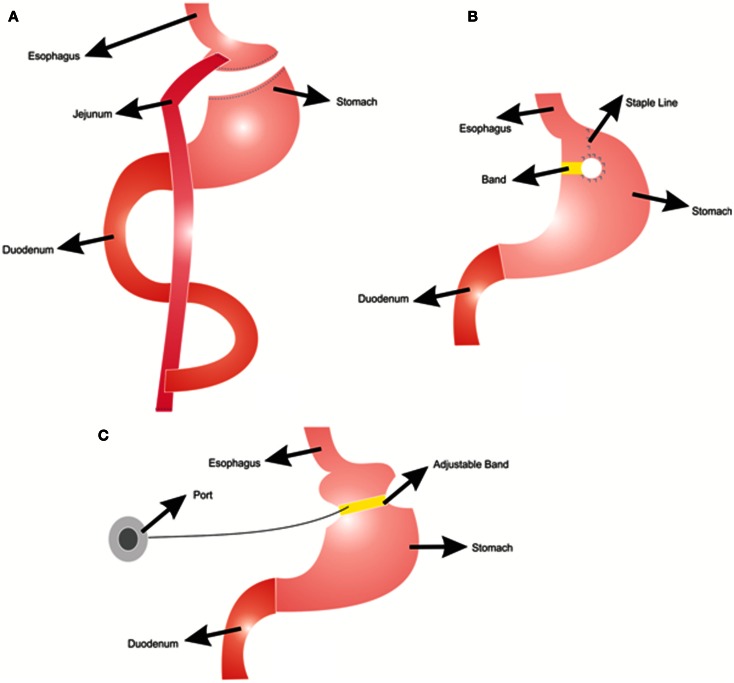
**Bariatric surgery procedures; (A) Roux-n-Y Gastric Bypass, (B) Vertical Gastroplasty, (C) Adjustable Gastric Banding**.

Different types of bariatric surgeries are used for reducing body weight with various success rates (Franco et al., 2011). In Roux-en-Y gastric bypass, the stomach is divided into two parts: a small proximal pouch (15–20 ml) and a large distal pouch (Figure 1A). The small proximal pouch is attached to the proximal jejunum, bypassing the large distal gastric pouch and duodenum (Jaunoo and Southall, 2010). In this procedure energy intake is restricted by the small volume of the stomach pouch (Arceo-Olaiz et al., 2008; Yan et al., 2008). Moreover, bypassing the duodenum decreases the digestion and absorption of food. In vertical banded gastroplasty, the proximal stomach is stapled vertically, allowing food draining from the proximal pouch to the distal pouch with the outlet reinforced with a mesh collar to prevent the enlargement of proximal pouch and staple line failures (Figure 1B) (Franco et al., 2011). This procedure decreases the energy intake due to reduced stomach size. However, staple line failures are very common problems that may lead to regaining of the lost weight (van Hout et al., 2007). In gastric banding, a cuff band is used to section the stomach into proximal and distal parts (Figure 1C). In the adjustable form, there is an inflatable balloon in the cuff band. A reservoir can be placed under the skin and band size can be adjusted by inflating the balloon from this reservoir (Camerini et al., 2004; Spivak et al., 2005; Picot et al., 2009). The Roux-en-Y gastric bypass procedure is more effective for weight loss than the vertical banded gastroplasty and the adjustable gastric banding (Picot et al., 2009).

## Inflammation in Obesity

Adipose tissue is considered as an endocrine organ, which consists of<50% adipocytes and>50% stromal vascular fraction that contains blood cells, endothelial cells, adipose-tissue precursors and stem cells, macrophages, and other immune cells (Wang and Nakayama, 2010). As an endocrine organ, the adipose-tissue produces and secretes several hormones and cytokines that play important roles in carbohydrate and lipid metabolism, inflammation, blood coagulation, as well as satiety, and hunger signaling (Hajer et al., 2008; Qasim et al., 2008; Wang and Nakayama, 2010). These cytokines, also known as adipocytokines or adipokines, are either secreted by adipocytes and/or by the stromal vascular fraction, especially macrophages, as summarized in Table 1 (Wang and Nakayama, 2010; Gomez-Illan et al., 2012; Kalupahana et al., 2012). Adipokines can function as classical cytokines, growth factors and proteins that are involved in blood pressure regulation, vascular homeostasis, and lipid and glucose metabolisms (Trayhurn, 2007).

**Table 1:** **Obesity-associated changes in the levels of common cytokines secreted by adipocytes and macrophages**.

Cytokines	Changes	Reference
**ADIPOCYTE SECRETIONS**		
Leptin	↑	Oswal and Yeo (2010)
Adinopectin	↓	Asayama et al. (2003)
Visfatin	↑	Sandeep et al. (2007a)
Vaspin	↑	Li et al. (2008)
Apelin	↑	Boucher et al. (2005)
FABP-4	↓	Queipo-Ortuno et al. (2012)
Perilipin	↓	Wang et al. (2003)
RBP-4	↑	Wolf (2007)
Resistin	↑	Piestrzeniewicz et al. (2008)
Lipocalin 2	↑	Catalan et al. (2009)
**MACROPHAGE SECRETIONS**		
IL-6	↑	Cesari et al. (2005)
TNF-α	↑	Tzanavari et al. (2010)
HGF	↑	Bell et al. (2006)
IL-10	↓	Blüher et al. (2005)
IL-18	↑	Leick et al. (2007)
PAI-1	↑	Cesari et al. (2005)
CRP	↑	Park et al. (2005)
MCP-1	↑	Panee (2012)
VEGF	↑	Garcia de la Torre et al. (2008)

FABP-4, Fatty Acid Binding Protein-4; RBP-4, Retinol Binding Protein-4; IL-6, Interleukin 6; TNF-α, Tumor necrosis factor-alpha; HGF, Hepatocyte Growth Factor; IL-10, Interleukin 10; IL-18, Interleukin 18; PAI-1, Plasminogen activator inhibitor-1; CRP, C-reactive Protein; MCP-1, Monocyte Chemoattractant Protein-1; VEGF, Vascular Endothelial Growth Factor.

In obesity, expansion of adipose-tissue causes hypoxia and stress, leading to necrosis of adipocytes. More than 90% of macrophages in white adipose tissues of animals are localized to dead adipocytes. The “Crown-Like Structure” (Yudkin, 2007) describes necrotic cells with impaired cell integrity and lipid droplets (Mosser and Edwards, 2008) and the surrounding macrophages that serve as scavengers of cell debris and lipid droplets in the necrotic cells.

There are two types of macrophages in white adipose tissues: proinflammatory M1 type and anti-inflammatory M2 type (Rull et al., 2010). M1 type macrophages are mainly recruited and induced by proinflammatory cytokines produced by expanded adipose tissue. After M1 type macrophages infiltrate into the adipose tissue, they secrete more proinflammatory cytokines and produce reactive oxygen species (ROS), which can recruit more macrophages and amplify the inflammatory response. M2 type macrophages are adipose-tissue resident macrophages (Rull et al., 2010). These macrophages are also called as alternatively activated macrophages because they are activated by interleukin (IL)-4. M2 type macrophages secrete anti-inflammatory cytokines and have mannose receptors, scavenger receptors and distinct integrins, which lead to anti-inflammatory functions (Mosser and Edwards, 2008; Rull et al., 2010). With stress and hypoxia in expanded adipose-tissue during obesity, M2 type macrophages lose their IL-4 receptor expression and IL-4-mediated anti-inflammatory functions, and instead are differentiated into M1 type proinflammatory macrophages (Rull et al., 2010). Besides IL-4, decreased expression of IL-10, Ym1, Arginase-1, and increased expression of tumor necrosis factor-alpha (TNF-α) and inducible nitric oxide synthetase (iNOS) induce the switch of adipose-tissue macrophages from a M2 to M1 phenotype.

This switch increases the number of proinflammatory macrophages, resulting in increased production of cytokines and chemokines including monocyte chemoattractant protein (MCP)-1, MCP-2, Regulated upon Activation, Normal T cell Expressed and Secreted (RANTES), and chemokine receptors like CCR2 and CCR5 (Wang and Nakayama, 2010). These inflammatory chemokines further increase macrophage infiltration to the adipose tissue (Malavazos et al., 2005; Trayhurn, 2005).

Kanda et al. found that macrophages and endothelial cells in rat adipose tissue can secrete MCP-1. This chemokine and its receptor CCR2 direct the migration of monocytes into the adipose tissue. Subsequent exposure of the monocytes to macrophage colony-stimulating factor causes differentiation to macrophages, which can secrete more MCP-1 (Kanda et al., 2006). In another study, obese subjects had significantly higher plasma MCP-1 levels than lean subjects, and increased plasma MCP-1 levels were positively associated with insulin resistance (Catalan et al., 2007). In expanded adipose tissue, M1 type macrophages secrete not only MCP-1, but also other proinflammatory cytokines including TNF-α, IL-6, IL-1, IL-1β, and IL-8, which can amplify inflammatory responses (Coppack, 2001; Zeyda and Stulnig, 2009).

## The Roles of Inflammation on Insulin Signaling

**Figure 2:**
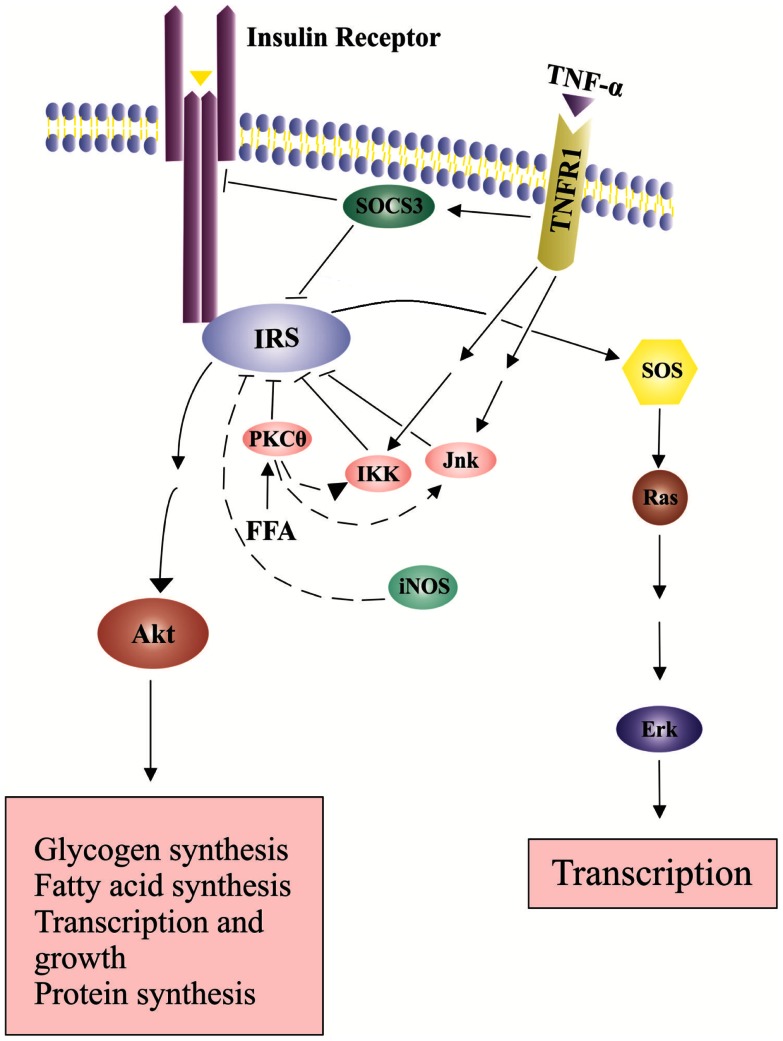
**Effects of TNP-α on insulin signaling pathways**. Insulin signals tyrosine phosphorylation of IRS and activates SOS (Son of Sevenless) and the downstream Erk pathway, leading to transcription and growth. In addition, insulin-stimulated Akt phosphorylation leads to glucose uptake, glycogen synthesis, protein synthesis, cell survival and gene transcription. The binding of TNF-α to its receptor increases SOCS-3 expression and activates serine kinases including INK and IKK, which inhibit IRS tyrosine phosphorylation and activation.

The major regulators in the insulin signaling pathway are insulin receptor substrates (IRSs). The binding of insulin to its receptor leads to tyrosine phosphorylation of the receptor. This phosphorylation is recognized by the IRS family that has 6 member proteins, IRS1 to IRS6. IRS1 plays an important role in transmitting signals from the insulin receptor on cell membrane to intracellular phosphatidiylinositol-3-kinase (PI3K)/Akt and extracellular signal-regulated kinases (Erk)/mitogen-activated protein kinase (MAPK) pathways (Cai et al., 2003; Tarantino and Caputi, 2011). Under normal circumstance, the binding of insulin to the insulin receptor induces a conformational change of the receptor, which leads to autophosphorylation of specific tyrosine residues in the cytoplasmic domains of the receptor and further recruitment of adapter proteins IRSs (Nieto-Vazquez et al., 2008). After interacting with IRSs, PI3K is activated. PI3K lipid products can further recruit and activate Akt through phosphorylation on threonine 308 (T308) (Nieto-Vazquez et al., 2008). Akt is an important component of insulin signaling pathways. IRS1 tyrosine phosphorylation leads to a second wave of phosphorylation in the Akt protein. Phosphorylated Akt then initiates more phosphorylation reactions and eventually leads to glucose uptake, glycogen synthesis, protein synthesis, cell survival and gene transcriptions (Siddle, 2011) (Figure 2). Tyrosine phosphorylation is required for IRS1 activation and insulin sensitization, which are blocked by serine phosphorylation on IRS1.

Increased production of TNF-α in expanded adipose-tissue decreases insulin sensitivity (Fantuzzi, 2008). TNF-α binds to TNF receptor-1 (TNFR1) and activates serine kinases including c-Jun N-terminal kinase (JNK), serine kinases inhibitor κB kinase (IKK) and S6 kinase (S6K). These serine kinase can cause serine phosphorylation of IRS1, block activation of downstream signaling molecules, and further decrease insulin sensitivity (Fain, 2010) (Figure 2). JNK and IKK are important components of the activator protein-1 (AP-1) and nuclear factor kappa B (NF-kB) cascade, respectively (Dhanasekaran and Johnson, 2007; Israel, 2010). NF-kB cascade includes a series of transcription factors that are sequestered by inhibitor of kappa B (IkB) proteins in the cytoplasm under normal conditions. IkB kinase (IKK), an important kinase in the NF-kB cascade, can phosphorylate and inactivate IkB proteins, and activate NF-kB (Israel, 2010). Proinflammatory cytokines like TNF-α can bind to its cell membrane receptor (TNFR1), activate IKK, further phosphorylate and inactivate IkB proteins (Israel, 2010). Subsequently, active NF-kB can translocate into the nucleus, bind on the promoter regions of cytokine genes, and stimulate proinflammatory cytokine expression (Ndisang, 2010). TNF-α also increases the expression of cytokine signaling (SOCS)-1 and SOCS-3 proteins, which can inhibit tyrosine phosphorylation of IRS1 and induce ubiqutinylation and degradation of IRS1 (Figure 2). The expression of SOCS-1 and SOCS-3 is also stimulated by IL-6. SOCS-3 blunts hepatocyte insulin signaling by binding to insulin receptors and leading to degradation of IRS proteins (Rasouli and Kern, 2008; Balistreri et al., 2010). In the absence of SOCS-3, IL-6 can have somewhat anti-inflammatory properties (Johnston and O’Shea, 2003). Increased plasma IL-6 concentrations are associated with insulin resistance (Charles et al., 2011). In addition, nitric oxide (NO), an endogenous signaling molecule produced by iNOS, can reduce Akt activity (Rasouli and Kern, 2008).

TNF-α and IL-6 can also alter the protein expression of peroxisome proliferator-activated receptor (PPAR)-γ in adipocytes (Leff et al., 2004; Tilg and Moschen, 2008; Zhou et al., 2008; Fernandez-Veledo et al., 2009). PPARγ is an anti-inflammatory nuclear protein with insulin sensitizing functions (Fernandez-Veledo et al., 2009). PPARγ blunts inflammatory responses and stimulates a switch of adipose-tissue macrophages from a M1 to M2 phenotype (Zeyda and Stulnig, 2009). PPARγ can inhibit the NF-kB signaling pathway, which is an insulin desensitizing pathway because it activates serine kinases and up-regulates the production of proinflammatory cytokines like TNF-α and IL-6 (Sidhu et al., 2003). However, JNK and IKK serine kinases can induce NF-kB activation and further increase TNF-α and IL-6 production, resulting in the suppression of PPARγ in adipocytes (O’Rourke, 2009). This suppression blunts PPARγ insulin sensitizing functions and leads to insulin resistance via the up-regulated NF-kB signaling pathway (Sidhu et al., 2003). Other than the serine kinases, saturated fatty acids can activate the NF-kB signaling pathway by binding to toll-like receptor 2 (TLR2) and 4 (TLR4) in adipocytes. TLR2 and TLR4 are important receptors of the immune system. Saturated fatty acids can bind to TLRs as their ligands and increase proinflammatory cytokine production (Jagannathan et al., 2010). Activation of TLR2 and TLR4 eventually contributes to insulin resistance via activating the NF-kB signaling pathway and increasing proinflammatory cytokine production (Monteiro and Azevedo, 2010).

C-reactive protein (CRP), an acute phase protein, is primarily synthesized in the liver and plays an important role in regulating the innate immune system (Eisenhardt et al., 2009). CRP aggravates the inflammatory status and leads to systemic inflammation (Wang and Nakayama, 2010). Plasma CRP levels are correlated with circulating levels of other inflammatory biomarkers (Kones, 2010). There is also a strong positive correlation between plasma CRP levels and insulin resistance (Pradhan et al., 2003; Pfutzner and Forst, 2006). Hepatic synthesis of CRP is driven at the transcriptional level by IL-6, which is mainly secreted by macrophages, T cells, and adipocytes (Pfutzner and Forst, 2006; Pfutzner et al., 2006). Since CRP mRNA levels rise in expanded adipose tissue, it has been proposed that adipose cells also have some ability to synthesize CRP (Memoli et al., 2007).

## The Changes of Novel Adipokines after Bariatric Surgery and Their Relationship to Insulin Resistance

Other than the classic cytokines and chemokines such as TNF-α, MCP-1, and IL-6, adipocytes also secrete leptin, adiponectin, visfatin, resistin, omentin, apelin, vaspin, perilipin, adipsin, and retinol binding protein-4. All these adipokines can contribute to systemic inflammation and pathogenesis of obesity-associated complications (Balistreri et al., 2010). An evidence-based review by Spector and Shikora (2010) attributed the bariatric surgery-induced insulin sensitization to surgery-specific, metabolic effects on glucose homeostasis that are independent of weight loss. However, the underlying mechanisms are not yet understood. This review article focuses on novel adipokines including visfatin, resistin, apelin, vaspin, and retinol binding protein-4 (RBP-4) and addresses their changes after bariatric surgery and their relationship to insulin resistance (Table 2).

**Table 2:** **The origin and roles of some novel adipokines in inflammation and insulin signaling and their responses to obesity, insulin resistance and bariatric surgery**.

Adipokine	Origin	Roles in inflammation	Roles in insulin signaling	Response to obesity	Response to insulin resistance	Response to bariatric surgery
Visfatin	Visceral adipose tissue, lymphocytes, monocytes, neutrophils, hepatocytes	Inflammation stimulates its production (Kim et al., 2007)	Insulin-mimetic effects via binding to the insulin receptor and further activating IRSs, PI3K and Akt (Fukuhara et al., 2005)	Inconclusive (Dogru et al., 2007; Botella-Carretero et al., 2008)	Inconclusive (Dogru et al., 2007; Alghasham and Barakat, 2008; Retnakaran et al., 2008)	Elevated (Botella-Carretero et al., 2008)
Resistin	Visceral adipose tissue	Proinflammatory effects via activating NF-kB (Maenhaut and Van de Voorde, 2011)	Insulin desensitizing effects via activating NF-kB and SOCS-3 (Steppan et al., 2005)	Elevated (Moschen et al., 2009)	Inconclusive (Iqbal et al., 2005, Sentinelli et al., 2002; Lee et al., 2003)	Inconclusive (Iqbal et al., 2005; Marantos et al., 2011)
Apelin	Central nervous system, adipose tissue, heart, kidneys, liver, brain	Unknown	Insulin sensitizing effects via activating AMPK, PI3K and Akt (Attane et al., 2011)	Elevated but not entirely due to obesity (Boucher et al., 2005; Castan-Laurell et al., 2011)	Elevated (Boucher et al., 2005; Soriguer et al., 2009; Dray et al., 2010; Yu et al., 2012)	Decreased (Soriguer et al., 2009)
Vaspin	Visceral adipose tissue, subcutaneous adipose tissue	Unknown	Insulin sensitizing effects (Kloting et al., 2006)	Elevated (Youn et al., 2008; Chang et al., 2010)	Elevated (Ye et al., 2009; Kempf et al., 2010)	Decreased (Chang et al., 2010; Handisurya et al., 2010)
RBP-4	Adipose tissue, liver	Unknown	Inhibits PI3K and IRS activation, and GLUT4 protein expression (Wolf, 2007; Esteve et al., 2009)	Mostly elevated (Yao-Borengasser et al., 2007; Bajzova et al., 2008; Gomez-Ambrosi et al., 2008)	Elevated (Graham et al., 2006; Esteve et al., 2009)	Decreased (Janke et al., 2006; von Eynatten et al., 2007; Bajzova et al., 2008)

### Visfatin

Visfatin is produced and secreted mainly by adipocytes in visceral adipose tissue (Tokunaga et al., 2008). Visfatin is also produced by a variety of cells including lymphocytes, monocytes, neutrophils, and hepatocytes (Kukla et al., 2011). This peptide adipokine produces insulin-mimetic effects by binding to the insulin receptor. The binding and further activation of the insulin signaling pathway can stimulate glucose uptake and increase insulin sensitivity (Fukuhara et al., 2005; Tilg and Moschen, 2006; Sun et al., 2007; Tokunaga et al., 2008; Fain, 2010). Visfatin and insulin bind to the different sites of the same insulin receptor (Tilg and Moschen, 2006). This difference in binding sites allows visfatin and insulin to work non-competitively. Similar to insulin, visfatin stimulates the phosphorylation of IRS1, IRS2, PI3K binding to IRSs, and activation of Akt and further promotes insulin sensitivity (Fukuhara et al., 2005; Adya et al., 2008a; Tan et al., 2009). Some studies suggested that NF-kB, JNK, and AP-1 upregulate visfatin production (Kim et al., 2007, 2008; Adya et al., 2008b; McGee et al., 2011).

There are many inconclusive data regarding the relationship between serum visfatin levels and body fat percentage or insulin resistance. Several studies suggest that blood visfatin levels are significantly related to type 2 diabetes or insulin resistance, but not to body fat percentage or BMI (Sandeep et al., 2007a; Palin et al., 2008; Retnakaran et al., 2008). One study compared plasma visfatin levels between type 2 diabetes subjects and non-diabetic healthy subjects and found that type 2 diabetes subjects had higher serum visfatin levels than non-diabetic healthy subjects (Sandeep et al., 2007b). However, this positive correlation between serum visfatin levels and diabetes status was no longer significant after adjusting for anthropometrics such as BMI and waist circumference (Dogru et al., 2007; Alghasham and Barakat, 2008; Retnakaran et al., 2008). In contrast, other studies reported that serum visfatin levels were significantly associated with obesity even after adjusting for age, sex, and diabetes (Fukuhara et al., 2005; Sandeep et al., 2007b). Moreover, serum visfatin levels were more associated with visceral fat mass than subcutaneous fat mass and appear to vary according to the body fat composition (Sun et al., 2007; Botella-Carretero et al., 2008). More studies are required to investigate their relationship.

Surprisingly, plasma visfatin concentrations were elevated after bariatric surgery (Krzyzanowska et al., 2006; Garcia-Fuentes et al., 2007; Botella-Carretero et al., 2008; Friebe et al., 2011). In one study of 53 severely obese persons who underwent biliopancreatic diversion (*n* = 38) and gastric bypass (*n* = 15), plasma visfatin levels were significantly increased 7 months after the surgery, and they were positively correlated with the percentage of waist circumference reduction (Garcia-Fuentes et al., 2007). In another report, there was an almost 50% increase in plasma visfatin levels post-compared to pre-biliopancreatic or laparoscopic bariatric surgery. In this study, multiple regression analysis showed that weight loss, diabetic status, and waist circumference changes were the main contributors to the increased serum visfatin levels (Botella-Carretero et al., 2008). Overall, these results indicate that plasma visfatin levels tend to be increased after weight loss surgeries (Garcia-Fuentes et al., 2007; Botella-Carretero et al., 2008). Given the well documented insulin-mimetic effect visfatin, the reason for the increased visfatin levels after weight loss is unclear, but may indicate a role for visfatin in improved insulin sensitivity after weight loss surgeries.

### Resistin

Resistin is mainly secreted by adipocytes and macrophages found in visceral adipose tissue. Increased plasma resistin levels lead to over-production of glucose from the liver and inhibit preadipocyte differentiation; however the precise mechanisms are still unclear (Steppan et al., 2001; Steppan and Lazar, 2002; Wolf, 2004). In rats, resistin induces severe hepatic insulin resistance and increases glucose production (Piestrzeniewicz et al., 2008). In human studies the role of resistin in insulin resistance and glucose metabolism is inconclusive (Lee et al., 2003; Qasim et al., 2008). The proinflammatory effects of resistin were attributed to its ability to activate NF-kB signaling pathway and subsequently increase the production of proinflammatory cytokines including TNF-α and IL-6, both of which can impair insulin signaling pathways and lead to insulin resistance (Zeyda and Stulnig, 2009; Singla et al., 2010; Maenhaut and Van de Voorde, 2011). In addition, resistin can activate SOCS-3 protein, which can lead to insulin resistance (Steppan et al., 2005). Although some studies suggest a relationship between resistin and glucose metabolism, others disagree (Sentinelli et al., 2002; Lee et al., 2003; Iqbal et al., 2005). Moreover, several studies reported that increased plasma resistin levels were associated with increased BMI (Filkova et al., 2009; Moschen et al., 2009).

Effects of bariatric surgery on plasma resistin levels are inconclusive. Some studies found that plasma resistin levels were significantly decreased 12 months after bariatric surgery with>10% loss of the excess body weight (Edwards et al., 2010; Jankiewicz-Wika et al., 2011; Marantos et al., 2011). These studies also reported that plasma resistin levels were positively correlated with insulin resistance and glucose intolerance (Moschen et al., 2009; De Luis et al., 2010; Jankiewicz-Wika et al., 2011; Marantos et al., 2011). Other studies showed no difference in plasma resistin levels after significant weight loss caused by bariatric surgery (Wolfe et al., 2004; Iqbal et al., 2005). More studies are required to investigate the relationship between plasma resistin levels and weight loss.

### Apelin

Apelin is a recently discovered peptide that is secreted by diverse tissues including central nervous system, adipose, and many other peripheral tissues, such as heart, kidneys, liver, and brain. Apelin is a binding ligand for the orphan G-protein–coupled receptor APJ (Yamamoto et al., 2011). A common 77-amino-acid precursor produces three bioactive apelin, which contain 13 amino acids (apelin-13), 17 amino acids (apelin-17), or 36 amino acids (apelin-36), respectively (Beltowski, 2006; Reinehr et al., 2011). Animal studies showed that apelin-deficient mice are insulin resistant and develop hyperinsulinemia, which can be reversed by administration of exogenous apelin (Yue et al., 2010). Administration of apelin to diabetic (db/db) obese mice can increase glucose uptake and elevate insulin sensitivity (Dray et al., 2008; Yue et al., 2010). Apelin stimulates PI3K/Akt phosphorylation-dependent GLUT 4 translocation, therefore increases glucose uptake by adipocytes (Lee et al., 2006; Zhu et al., 2011). Furthermore, apelin secretion can activate AMP-activated protein kinase (AMPK) pathway and leading to an insulin sensitizing effect (Kadoglou et al., 2010; Attane et al., 2011).

Apelin expression and secretion are increased during adipocyte differentiation and are regulated nutritionally and hormonally (Boucher et al., 2005; Dray et al., 2008). Type 2 diabetes subjects have significantly higher plasma apelin levels than non-diabetic healthy subjects. The increase in plasma apelin levels may be a result of compensatory response to insulin resistance (Boucher et al., 2005; Soriguer et al., 2009; Dray et al., 2010). Obese subjects have significantly higher plasma apelin levels than lean subjects (Boucher et al., 2005; Heinonen et al., 2005; Castan-Laurell et al., 2011). Soriguer et al. (2009) demonstrated that the plasma apelin levels were increased only in obese and diabetic subjects, not in obese and non-diabetic subjects, as compared with control subjects. This indicates that obesity and body fat mass may not be the main factors altering circulating apelin levels (Boucher et al., 2005), and insulin resistance may be more important than obesity in increasing plasma apelin levels in humans. Although there is a strong positive correlation between plasma apelin levels and TNF-α expression in expanded adipose tissue, the role of apelin in regulating inflammatory response is still not clear (Boucher et al., 2005; Daviaud et al., 2006).

Plasma apelin levels can vary according to the diabetic status after weight loss following the bariatric surgery. In a study, diabetic morbidly obese patients had significantly higher plasma apelin levels than non-diabetic non-obese healthy subjects. After bariatric surgery, plasma apelin levels in morbidly obese subjects with impaired blood glucose were significantly decreased (Soriguer et al., 2009). Krist et al. (2013) also demonstrated that bariatric surgery dramatically decreased the apelin expression in adipose tissues and serum apelin levels, which significantly correlated with improved insulin sensitivity. This correlation is independent of BMI changes. More studies are required to investigate the underlying mechanisms.

### Vaspin

Vaspin is a member of the serine protease inhibitor family and is also known as visceral adipose-tissue derived serpin. But human subcutaneous adipose tissue, liver, stomach, and rodent hypothalamus also express vaspin (Lee et al., 2011). Vaspin is also expressed in adipose tissues in Otsuka Long-Evans Tokushima rats, which are used as an animal model for studying type 2 diabetes (El-Mesallamy et al., 2011). Studies suggest that vaspin may have important roles in obesity and insulin resistance (Li et al., 2011). Administration of vaspin to obese mice improved glucose tolerance and elevates insulin sensitivity (Hida et al., 2005; Wada, 2008). However, the underlying mechanisms of improving insulin sensitivity are not known.

Some studies demonstrate that diabetic subjects have higher serum vaspin levels than non-diabetic subjects (Ye et al., 2009; Kempf et al., 2010). Lean subjects have lower serum vaspin levels than overweight subjects (Suleymanoglu et al., 2009). Others reported a positive association between blood vaspin levels and BMI in obesity (Youn et al., 2008; Chang et al., 2010; Bluher, 2012). Blood vaspin levels and BMI correlation is strong in type 2 diabetes or insulin resistant patients. This indicates that obesity-induced insulin resistance may be more important than BMI in regulating circulating vaspin levels (Youn et al., 2008; Chang et al., 2010; Kempf et al., 2010). Since vaspin can improve glucose tolerance and insulin sensitivity, the increase in plasma vaspin levels may be a result of compensatory response to insulin resistance.

Weight loss decreases plasma vaspin levels in humans (Kloting et al., 2006; Li et al., 2008; Youn et al., 2008). Weight loss after bariatric surgery significantly decreases plasma vaspin levels, which also correlates with decreased plasma insulin levels and improved insulin sensitivity (Chang et al., 2010; Handisurya et al., 2010). This decrease might be a compensatory mechanism associated with weight loss and insulin sensitivity. Even though these findings suggest that vaspin may have some roles in regulating glucose metabolism and insulin signaling pathways, the mechanisms are not yet understood (Kloting et al., 2006; Youn et al., 2008).

### Retinol binding protein-4

Retinol binding protein-4 (RBP-4) is secreted predominantly by adipocytes and hepatocytes. In plasma, RBP-4 is the carrier protein of retinol and appears to relate to glucose metabolism and insulin sensitivity (Esteve et al., 2009). Increased RBP-4 levels lead to reduced glucose uptake by muscle cells through inhibiting PI3K signaling pathway and serine phosphorylation of IRS1 (Wolf, 2007), followed by decreased insulin sensitivity. Some studies demonstrated an inverse correlation between glucose transporter 4 (GLUT4) protein expression and plasma RBP-4 levels (Graham et al., 2006; Esteve et al., 2009; Toruner et al., 2010). Decreased GLUT4 expression is accompanied by increased RBP-4 secretion in the adipose tissue of obese subjects and reduced GLUT4 expression can be caused by increased RBP-4 secretion (Yang et al., 2005). In liver, RBP-4 stimulates the expression of phosphoenolpyruvate carboxykinase (PEPCK) enzyme, a gluconeogenic enzyme that is stimulated by glucagon and inhibited by insulin (Quinn and Yeagley, 2005), leading to impaired insulin signaling in hepatocytes (Yang et al., 2005).

Most but not all of the findings suggest that plasma RBP-4 levels are positively associated with body fat percentage and insulin resistance (Janke et al., 2006; von Eynatten et al., 2007; Yao-Borengasser et al., 2007; Bajzova et al., 2008; Gomez-Ambrosi et al., 2008). Obese subjects have higher plasma RBP-4 levels than lean subjects. Plasma RBP-4 levels are positively correlated with insulin resistance in subjects with obesity, impaired glucose tolerance, or type 2 diabetes (Graham et al., 2006). RBP-4 levels were significantly decreased following weight loss after a bariatric surgery (Haider et al., 2007; Gomez-Ambrosi et al., 2008; Tschoner et al., 2008). Barazzoni et al. (2011) showed that high plasma RBP-4 levels was correlated with high systemic inflammatory responses in non-obese, non-diabetic patients with chronic kidney disease (But the correlation was independent of RBP-4 expression in adipose tissue. More studies are required to investigate the roles of RBP-4 in inflammation.

## Other Changes in Gastrointestinal System after Bariatric Surgery

After a meal, gastrointestinal system secretes incretins including glucose dependent insulinotropic polypeptide (GIP) and glucagon-like peptide 1 (GLP-1), which stimulate post-prandial insulin secretion (Holst, 2013; Holst and Deacon, 2013). GIP is secreted by duodenal K cells and GLP-1 is secreted by ileal L cells (Holst, 2013). These incretins have insulinotropic effects, furthermore; GLP-1 delays gastric emptying and decreases appetite leading to increased weight loss and insulin sensitivity (Flint et al., 2001; Laferrère, 2011). GLP-1 is increased after Roux-en-Y gastric bypass and biliopancreatic diversion (Näslund et al., 1997, 1998). Even though inconclusive findings on effects of the bariatric surgery on blood GIP levels are reported (Näslund et al., 1998; Laferrère, 2011, 2012), Laferrère et al. (2007) showed increased blood GIP levels 1 month and 1 year after Roux-en-Y surgery in patients with type 2 diabetes. Näslund et al. (1997) reported that the increase in blood GLP-1 and GIP levels is persistent for 20 years after duodenal jejunal bypass. More studies are required to investigate the effects of GLP-1 and GIP changes on inflammation in obese subjects following bariatric surgery.

There are two hypotheses trying to explain the increase in incretins secretion; foregut; and hindgut hypotheses (Strader et al., 2005; Strader, 2006; Pacheco et al., 2007; Hansen et al., 2011; de Luis et al., 2012). Foregut hypothesis suggests that bypassing duodenum and proximal jejunum increases incretin secretion which improves insulin sensitivity more than weight loss itself (Pacheco et al., 2007; Hansen et al., 2011; de Luis et al., 2012). However, research studies in Roux-en-Y gastric bypass and vertical sleeve gastrectomy which does not bypass duodenum or jejunum reported similar incretin secretion levels, therefore invalidating the foregut hypothesis (Chambers et al., 2011). Hindgut hypothesis suggests that instant stimulation of ileum by the nutrients causes the increase in blood GLP-1 levels (Strader et al., 2005; Strader, 2006). After Roux-en-Y gastric bypass, emptying of the stomach is fast and nutrients reach to ileum rapidly which might be the cause of increased GLP-1 secretion (Morínigo et al., 2006).

Bariatric surgery alters gut microbiota dramatically (Zhang et al., 2009; Furet et al., 2010; Clément, 2011; Kootte et al., 2012; Sweeney and Morton, 2013). Zhang et al. (2009) showed a positive association between Archaea and obesity. They found no Archaea in normal weight subjects and a decreased number in subjects who underwent gastric bypass surgery. Furet et al. (2010) demonstrated a decrease in Prevotellacaea during obesity, but the number of Prevotellacaea was rapidly increased after gastric bypass surgery. Zhang et al. showed obese subjects had more Bacteroidetes than lean subjects. Contrary to Furet et al. findings, Prevotellacaea, which is a subgroup of Bacteroidetes, was enriched in obese subjects. Furthermore, Ley et al. (2006) found no difference in the amount of Bacteroidetes between lean and obese subjects. These inconclusive data indicate that weight loss might be affecting subgroups differently. Effects of gut microbiota changes after bariatric surgery on inflammation, insulin resistance, and diabetes are not completely understood (Kootte et al., 2012). However, even though there might be positive effect of gut microbiota change on insulin sensitivity, this effect probably is overridden by possible malnutrition state after bariatric surgeries (Kootte et al., 2012).

## Conclusion and Future Directions

Obesity is a chronic low-grade inflammatory disease. In adipose tissue, both adipocytes and macrophages secrete a large number of hormones, proteins, cytokines, and chemokines, collectively called adipokines. These adipokines contribute to the pathogenesis of metabolic syndrome, insulin resistance, type 2 diabetes, and cardiovascular disease, most likely via regulating the inflammatory pathway mediated by TNF-α, IL-6, NF-kB, JNK, and IKK and insulin signaling pathway mediated by IRSs, PI3k/Akt, and SOCSs. Weight loss via surgeries dramatically alters levels of these adipokines and overall increases insulin sensitivity. However, despite ample evidence supporting the role of these adipokines in obesity and insulin resistance, mechanisms mediating their effects are for the most part still unclear. It is also unclear whether or how these adipokines mediate the effect of weight loss surgeries such as bariatric surgery on inflammation and insulin response. Further studies are required to better understand the relationship between those adipokines and insulin resistance and how these are modulated by bariatric surgery. Alteration in the adipokine profile primarily reflects changes in adipose-tissue production and secretion of these cytokines. Thus, additional studies are needed to determine whether reduced adipose-tissue inflammation following bariatric surgery is responsible for reduced systemic inflammation and improved insulin sensitivity. Furthermore, the development of animal models which recapitulate the bariatric surgery model will lead to a better understanding of mechanisms by which this surgery improves metabolic outcomes related to diabetes and obesity. It is especially important to understand whether the beneficial effects of bariatric surgery are solely related to weight loss, improved adipose-tissue inflammation and/or changes in gut physiology and endocrinology. In addition, with an adequately powered sample sizes for cross-sectional or case-control studies, the various types of adipose tissues, including subcutaneous, omental, and mesenteric adipose tissues, along with plasma and other tissues, can be collected from severely obese patients who undergo bariatric surgery. Finally, long term studies are critical to assess whether weight loss surgery and associated metabolic improvements are sustained over a long period of time and whether these are primarily related to weight loss or other beneficial effects of the bariatric surgery.

## Conflict of Interest Statement

The authors declare that the research was conducted in the absence of any commercial or financial relationships that could be construed as a potential conflict of interest.
